# Ambient temperature and coronary heart disease mortality in Beijing, China: a time series study

**DOI:** 10.1186/1476-069X-11-56

**Published:** 2012-08-21

**Authors:** Zhaoxing Tian, Shanshan Li, Jinliang Zhang, Jouni Jk Jaakkola, Yuming Guo

**Affiliations:** 1 Emergency Department of Peking University Third Hospital, Beijing, China; 2 School of Population health, The University of Queensland, Brisbane, Australia; 3 State Key Laboratory of Environmental Criteria and Risk Assessment & Environmental Standards Institute, Chinese Research Academy of Environmental Sciences, Beijing, China; 4 Center for Environmental and Respiratory Health Research, Institute of Health Sciences, University of Oulu, Oulu, Finland; 5 School of Public Health and Social Work, Queensland University of Technology, Brisbane, Australia; 6 School of Medicine, The University of Queensland, Brisbane, Australia

**Keywords:** Ambient temperature, Coronary heart disease, Mortality, Cold effect, Hot effect, Gender, Age

## Abstract

**Background:**

Many studies have examined the association between ambient temperature and mortality. However, less evidence is available on the temperature effects on coronary heart disease (CHD) mortality, especially in China. In this study, we examined the relationship between ambient temperature and CHD mortality in Beijing, China during 2000 to 2011. In addition, we compared time series and time-stratified case-crossover models for the non-linear effects of temperature.

**Methods:**

We examined the effects of temperature on CHD mortality using both time series and time-stratified case-crossover models. We also assessed the effects of temperature on CHD mortality by subgroups: gender (female and male) and age (age > =65 and age < 65). We used a distributed lag non-linear model to examine the non-linear effects of temperature on CHD mortality up to 15 lag days. We used Akaike information criterion to assess the model fit for the two designs.

**Results:**

The time series models had a better model fit than time-stratified case-crossover models. Both designs showed that the relationships between temperature and group-specific CHD mortality were non-linear. Extreme cold and hot temperatures significantly increased the risk of CHD mortality. Hot effects were acute and short-term, while cold effects were delayed by two days and lasted for five days. The old people and women were more sensitive to extreme cold and hot temperatures than young and men.

**Conclusions:**

This study suggests that time series models performed better than time-stratified case-crossover models according to the model fit, even though they produced similar non-linear effects of temperature on CHD mortality. In addition, our findings indicate that extreme cold and hot temperatures increase the risk of CHD mortality in Beijing, China, particularly for women and old people.

## Background

There is strong evidence that extreme temperatures (e.g., cold spells and heat waves) have significant impacts on health [1,2]. Studies have shown that vulnerable people (e.g., elderly, children, and people with chronic diseases) will be affected greatly by extreme temperatures [3]. Coronary heart disease (CHD) patients constitute one of the largest groups of susceptible people [4-6]. As the second leading cause of cardiovascular death in the Chinese population, CHD accounts for 22% of cardiovascular deaths in urban areas and 13% in rural areas in China [7]. There is evidence that the incidence of CHD is steadily increasing in China [8], but there is a gap in the knowledge about the effects of temperature on CHD mortality applicable for the Chinese population.

Season and long-term trends are considered as confounders in examining short-term effects of temperature on mortality. Time series models with a smooth function for time are commonly used to control for season and long-term trends [9-12]. The case-crossover study is an alternative design where seasonal effects and secular trends are taken into account by comparing exposure on a period shortly prior to or after the onset (hazard period) to reference periods in relatively small time windows (e.g., calendar month) [13,14]. This adjusts for season using a step-function rather than the smooth function used by time series [15]. Both methods have been used in estimating non-linear relations between temperature and mortality, but the two analytical approaches have not been compared in distributed lag non-linear models. Our objective was to assess the non-linear relations between temperature and CHD mortality using both time-series and time-stratified case-crossover analyses.

## Methods

### Data collection

Data on the daily numbers of deaths from CHDs and weather conditions were collected from urban areas in Beijing, China. Beijing is located in northern China, and has four distinct seasons, with cold, windy, dry winters, and hot, humid summers.

Data on the daily counts of death from CHDs were retrieved from the Death Classification System, Beijing Public Security Bureau from January 1, 2000 to December 31, 2011. We classified CHD mortality according to the International Classification of Diseases, 10^th^ revision (ICD-10: I20–I25). We stratified the deaths into groups by gender (women and men) and age (age > =65 and age <65).

We acquired meteorological data on daily mean temperature and relative humidity from the China Meteorological Data Sharing Service System. The data was monitored at a single monitoring station which is located at Daxing District (N39°48' E116°28') in the south eastern part of Beijing. There was no missing data for temperature and relative humidity. The reason why we used only one monitoring station’s temperature is that an unpublished study (Yuming Guo, in revision by Environmental Research) shows that time series model using one monitoring station’s temperature is equal to spatiotemporal model using spatial temperatures for assessing city-wide temperature effects on mortality.

### Data analysis

#### Time series analysis

We used a time series regression to examine the group-specific temperature-mortality relationship. We allowed for over-dispersion in CHD deaths using a quasi-Poisson function [16]. Many studies have reported that the temperature-mortality association is non-linear and might be delayed in time. Both cold and hot temperatures increase the risk of mortality, and they not only increase the risk of mortality on the current day, but also on several following days [17]. The lag days was fixed at 15 days, as most studies have shown that the association between cold temperature and mortality can last for weeks, while the association between hot temperature and mortality is usually acute with some mortality displacement [18]. Thus, we applied a distributed lag non-linear model (DLNM), by which the non-linear and delayed associations were modeled. We used a DLNM with 5 degrees of freedom natural cubic for temperature and with 4 degrees of freedom natural cubic for lags [19,20].

The time series model used a natural cubic spline with 7 degrees of freedom per year for time to control for seasonal pattern and long-term trend. We controlled for day of the week as an indicator variable. We controlled for relative humidity using the same DLNM as temperature.

#### Time-stratified case-crossover analysis

As an alternative, time-stratified case-crossover model was used to examine the group-specific temperature-mortality relationship. Time-stratified case-crossover model has been widely used to assess the effects of temperature (or air pollution) on mortality [21,22]. The case–crossover design is a special case of matched case–control study [23]; each case in the case-crossover study is used as their control. For the time series data on deaths and temperature, the case–crossover design compares temperatures on a hazard day prior to or after the onset of the event of interest (e.g., deaths) with temperatures on nearby reference days to examine whether the events are associated with temperature. Because reference days are selected close to the hazard days, seasonality is controlled by design [24,25]. The time-stratified case–crossover uses fixed and disjointed time strata (e.g., calendar month), so the overlap bias is avoided [26].

The conditional logistic regression used in case–crossover analysis is a special case of Poisson regression model [27,28]. Hence, we used a Poisson regression model with quasi-Poisson function to fit the time-stratified case–crossover design. We used calendar month as strata. For each death, the deceasing day was defined as “hazard day”. The same days of the week in the same calendar month were selected as “reference days”. Day of the week was controlled for by matching to avoid any potential confounding due to the strong weekly pattern in mortality. The same DLNM was used for temperature as time series analysis. Also, we used the same function for relative humidity.

For both time series and time-stratified case-crossover analyses, we plotted the estimated relative risks for each group. We calculated the relative risks at specific temperatures: the relative risks of death associated with an extremely cold temperature (−7.6°C, 1^st^ percentile of mean temperature) compared to 10^th^ percentile of temperature (−2.2°C); and associated with an extremely hot temperature (30.5°C, 99^th^ percentile of mean temperature) relative to 90^th^ percentile of temperature (27.0°C). These effect estimates were taken from the nonlinear temperature-mortality curves, so they reflect a portion of the true exposure–response curves [17].

We used Akaike information criterion for quasi-Poisson (Q-AIC) to assess which design (time series or case-crossover models) performed better. Sensitivity analyses were performed by changing the maximum lag from 15 to 30 days, the degrees of freedom for temperature, relative humidity and lags (3 to 6) and degrees of freedom (6 to 10 per year) for time in time series models. We also changed strata length (from 21 to 42 days) for time-stratified case-crossover models. All statistical tests were two-sided and *p*-values of less than 0.05 were considered statistically significant. The R software (version 2.15.0, R Development Core Team 2009) was used to fit all models, with the “dlnm” package to create the DLNM [20].

## Results

There were totally 26,460 CHD deaths in Beijing, China during 2000 to 2011, including 18,250 men and 19,358 people aged > =65 years. Table 1 shows the statistical summary for CHD mortality, mean temperature and relative humidity. The daily mean CHD mortality was 6, mean temperature 13.3°C, and relative humidity 52.7%.

**Table 1:** Summary statistics for coronary heart disease mortality and weather condition in Beijing, China during 2000 to 2011

**group**	**1%**	**10%**	**25%**	**50%**	**75%**	**90%**	**99%**	**Mean**	**SD**^***a***^
All	1	3	4	6	8	10	14	6	2.9
Male	0	1	2	4	6	7	11	4	2.3
Female	0	0	1	2	3	4	6	2	1.4
Age < 65	0	0	1	1	2	3	6	2	1.4
Age > =65	0	2	3	4	6	8	11	4	2.4
Mean temperature (°C)	−7.6	−2.2	2.5	14.7	23.8	27	30.5	13.3	11.2
Relative humidity (%)	14	24	36	54	69	79	91	52.7	20.3

^*a*^ Standard deviation.

**Figure 1:**
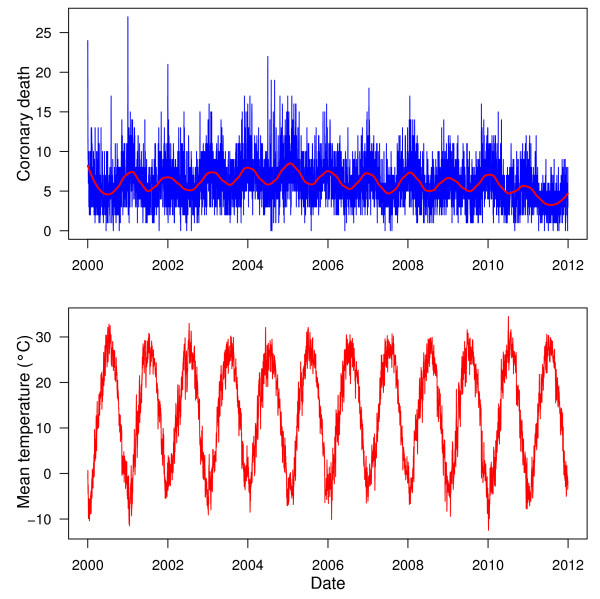
The time series of coronary heart disease mortality and mean temperature in Beijing, China during 2000 to 2011.

Figure 1 shows the time series of the CHD mortality and temperature in Beijing, China between 2000 and 2011. In general, there was a seasonal trend of CHD death, with higher mortality in winter than summer. There was a clear seasonal pattern for temperature.

**Figure 2:**
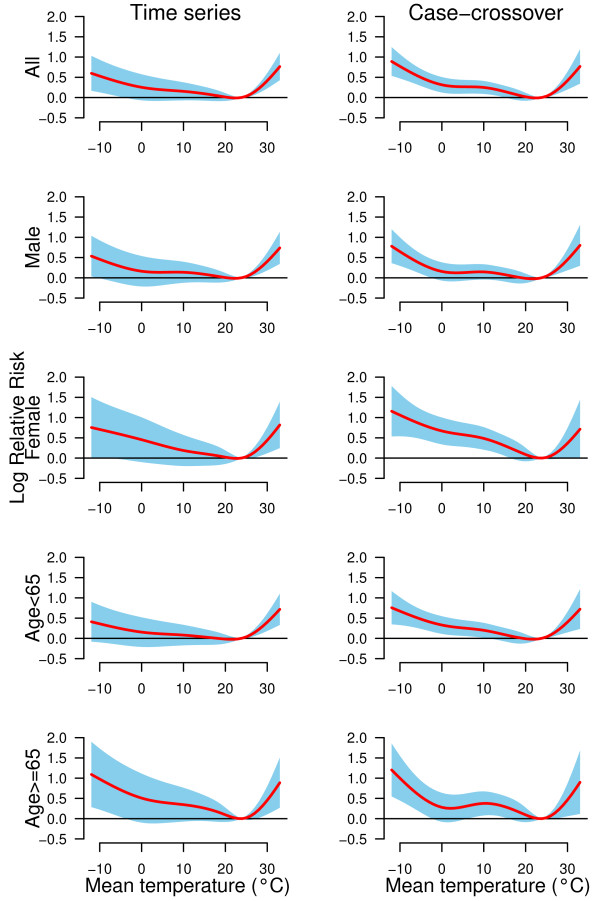
The non-linear effects of temperature on group-specific coronary heart disease mortality at lag 0–15, using time series and time-stratified case-crossover analyses with 5 degrees of freedom natural cubic spline for temperature.

Figure 2 shows the non-linear relations between temperature and group-specific CHD mortality using both time series and case-crossover analyses. The two models gave similar group-specific temperature-mortality relations. Both extreme cold and hot temperatures increased the risk of CHD mortality in all groups.

Table 2 Shows the effects of extreme cold and hot temperatures on CHD mortality by group, using both time series and case-crossover analyses. The case-crossover models produced higher effect estimates than time series models. However, time series models could be better to fit the data than case-crossover models (Table 3). Time series analyses show that the elderly and women were more sensitive to extreme cold and hot temperatures than young and men; the overall cold effect (−7.6°C versus −2.2°C) in CHD mortality risk was 1.16 (95% confidence interval: 1.04, 1.30); the overall hot effect (30.5°C versus 27.0°C) in CHD mortality risk was 1.38 (1.20, 1.60).

**Table 2:** The effects of extreme cold and hot temperatures on group-specific mortality from coronary heart disease over lags 0–15, using time series and case-crossover analyses with 5 degrees of freedom natural cubic spline for temperature

**Effects**	**group**	**Relative risk (95% CI)**	
		**Time series**	**Case-crossover**
Cold effect ^*a*^	All	1.16 (1.04, 1.30)*	1.29 (1.12, 1.48) *
	Male	1.15 (0.95, 1.39)	1.24 (0.97, 1.59) *
	Female	1.18 (1.03, 1.34) *	1.31 (1.11, 1.55) *
	Age < 65	1.12 (0.99, 1.27)	1.21 (1.02, 1.42) *
	Age > =65	1.29 (1.06, 1.58) *	1.49 (1.15, 1.93) *
Hot effect ^*b*^	All	1.38 (1.20, 1.60) *	1.39 (1.15, 1.67) *
	Male	1.37 (1.16, 1.62) *	1.37 (0.99, 1.88)
	Female	1.42 (1.11, 1.81) *	1.40 (1.12, 1.75) *
	Age < 65	1.35 (1.15, 1.59) *	1.36 (1.09, 1.68) *
	Age > =65	1.47 (1.13, 1.91) *	1.48 (1.05, 2.08) *

*P < 0.05.

^*a*^ 1^st^ percentile of temperature (−7.6°C) relative to 10^th^ percentile of temperature (−2.2°C).

^*b*^ 99^th^ percentile of temperature (30.5°C) relative to 90^th^ percentile of temperature (27.0°C).

**Table 3:** Akaike information criteria for quasi-Poisson (Q-AIC) values for the relationship between temperature and group-specific coronary heart disease mortality using time series and case-crossover models, with 5 degrees of freedom natural cubic spline for temperature and 4 degrees of freedom natural cubic spline for lags

**group**	**Q-AIC value**	
	**Time series**	**Case-crossover**
All	20606.4	21534.3
Male	18629.8	19597.2
Female	14714.4	15532.1
Age < 65	13963.6	14915.0
Age > =65	18955.8	19810.3

**Figure 3:**
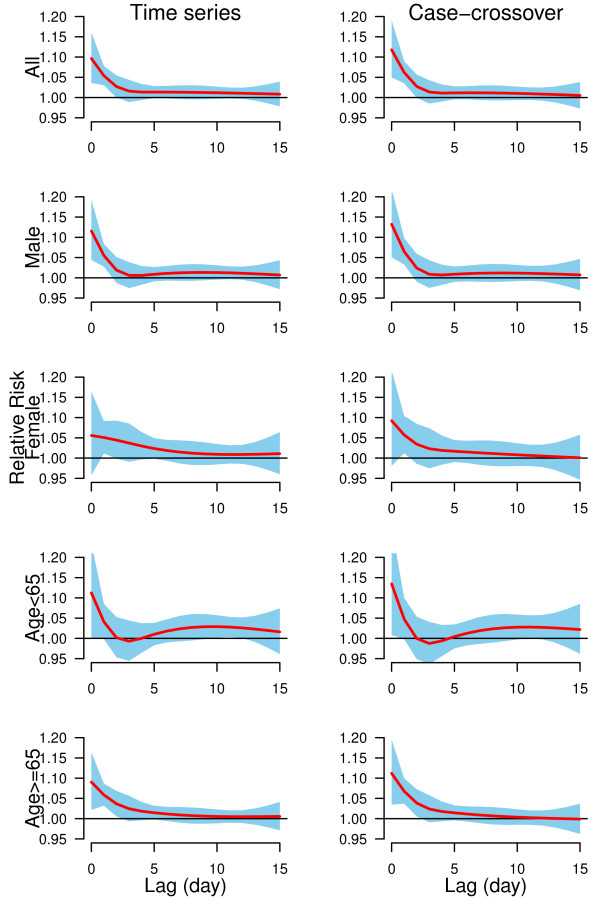
**The estimated hot effects associated with 99**^**th**^**percentile temperature (30.5°C) relative to 90**^**th**^**percentile of temperature (27.0°C) on group-specific CHD mortality over 15 days of lag, using time series and time-stratified case-crossover models with 5 degrees of freedom natural cubic spline for temperature and 4 degrees of freedom natural cubic spline for lag.**

**Figure 4:**
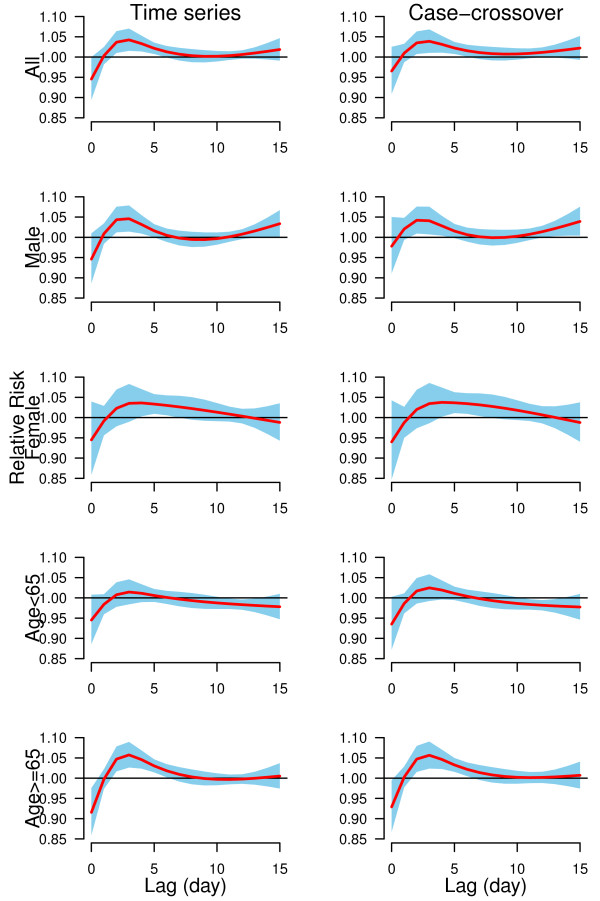
**The estimated cold effects associated with 1**^**st**^**percentile of temperature (−7.6°C) relative to 10**^**th**^**percentile of temperature (−2.2°C) on group-specific CHD mortality over 15 days of lag, using time series and time-stratified case-crossover models with 5 degrees of freedom natural cubic spline for temperature and 4 degrees of freedom natural cubic spline for lag.**

We plotted the lag structures for hot effects (Figure 3) and cold effects (Figure 4) up to 15 days. Figure 3 shows that the hot effects on all groups of CHD mortality were acute and short-term (lasted for 3 days). In general, cold effects were delayed by two days and lasted for five days (Figure 4). The change of lag from 15 to 30 days, and the degrees of freedom (3 to 6) for temperature, relative humidity and lags did not substantially influence the effect estimates. Time series models using degrees of freedom (6 to 10 per year) produced similar results as our results. Time-stratified case-crossover models using strata length (from 21 to 42 days) still gave similar estimates as our findings.

## Discussion

To our best knowledge, this is the first study to examine the effects of ambient temperature on CHD mortality in Beijing, China. This is also the first study using both time series and case-crossover models to examine the non-linear association between temperature and CHD mortality. Both models show that extreme cold and hot temperatures increased the risk of CHD mortality, but time series models performed better than case-crossover models to fit the data. The old people and women were more sensitive to extreme hot and cold temperatures than the young and men respectively.

Our results are consistent with previous findings. Kunst et al. [29] reported that the high CHD mortality in cold weather were largely attributable to exposure to cold temperatures, after controlling for the influences of influenza, air pollution and season. Danet et al. [30] found that the cold effects increased the risk of both CHD morbidity and mortality, with stronger effects in old people. Other studies reported that hot temperatures were associated with the high rates of CHD deaths [31,32]. Exposure to extreme hot temperatures induced an acute event in people with myocardial infarction or stroke [33].

We investigated both hot and cold effects over 15 days on CHD mortality. The hot effects were acute and short-term. Studies have shown that hot temperatures induce an acute event in people with myocardial infarction or stroke [33]. In general, cold effects were delayed and lasted about 5 days after the extreme cold days. Previous studies also reported similarly delayed cold effects on mortality [17].

No previous study has reported an association between temperature and CHD mortality in China. However some studies have provided evidence that cold and hot temperatures are related to the increased risks of non-accidental, cardiovascular, and respiratory mortality in Beijing [34], Tianjin [18], and Shanghai [35].

Our findings are biologically plausible for several reasons. Exposure to extreme cold temperatures is associated with an increase in blood pressure, blood cholesterol, heart rate, plasma fibrinogen, platelet viscosity and peripheral vasoconstriction, [36,37]. Exposure to extreme hot temperatures might induce dehydration, salt depletion and increased surface blood circulation, which can lead to a failure of thermoregulation [38]. Extreme hot temperatures may also be related to elevated blood viscosity, cholesterol levels and sweating thresholds [39].

Almost all the studies have indicated that the elderly are more sensitive to the impact of ambient temperature than the young [40,41], regardless of time periods, regions and methods [42]. The reason might be that the thermal regulation system weakens with aging, for example, skin sensory perception may diminish and thermal homeostasis may decline [43].

In this study, we found women to be more sensitive to extreme cold and hot temperatures than men in Beijing, China. There is evidence that women are more vulnerable to extreme cold and hot temperatures than men [44]. The reason might be that women have higher risks for ischemic, arrhythmic and blood pressure which are sensitive to the extreme hot and cold temperatures [45]. The other reason might be that women are older than men.

The time series and case–crossover analyses have been used widely to examine the effects of temperature (air pollution) on mortality in the past decade [24]. Many studies have compared these two models for the linear effects of temperature (or air pollution) on mortality, and results show that the two models are equal to examine the linear effects of temperature (or air pollution) on morality [21,22,24]. This study confirmed that the time series and case-crossover models gave the similar non-linear effect estimates for the temperature-mortality relation, even though time series models performed better than case-crossover models as judged by model fit (Q-AIC).

There are some limitations in this study. We used monitoring site data in exposure assessment. This might introduce exposure misclassification bias (Berkson error). However, Berkson error may reduce the power of the study, but it does not attenuate the risk estimates. An unpublished study (Yuming Guo, in revision by Environmental Research) also reports that one monitoring station’s temperature is enough to capture the city-wide temperature effects on mortality. We only used data from Beijing, China to examine the effects of temperature on CHD mortality, so the findings are difficult to generalize to other areas. Other studies in different countries using the same designs should be developed to confirm and detail the impacts of ambient temperature on CHD mortality. We did not control for air pollution, as these data were not available. However, some studies found that the temperature effects on mortality are robust after controlling for air pollution [17]. But future studies are still needed to look at this issue.

## Conclusions

Both time series and time-stratified case-crossover analyses indicate that extremely cold and hot temperatures increase the risk of CHD mortality in Beijing, China. Time series models were better to fit the data than case-crossover models according to the model fit. The women and old people were more sensitive to extreme cold and hot temperatures than men and young. These findings strongly suggest prevention of cold-/hot-related CHD event has a great public health potential. Adaptation measure such as appropriate heating and clothing in winter, using air condition in summer, as well as well-functioning alarm systems and emergency service could prevent substantial amount of CHD mortality. However, detailed studies are needed to identify and assess the most suitable adaptation measures for extreme cold and hot temperatures.

## Abbreviations

CHD: Coronary heart disease; DLNM: Distributed lag non-linear model; ICD-10: International classification of diseases, 10th revision; Q-AIC: Akaike information criterion for quasi-Poisson.

## Competing interests

The authors declare that they have no competing interests.

## Authors’ contributions

ZT and YG conceived and conducted the study design, and drafted the manuscript; YG performed data analysis; SL, JZ, JJ reviewed, edited, and revised the manuscript. All authors read and approved the final manuscript.
